# Impact of feeding dried distillers’ grains with solubles diet on microbiome and metabolome of ruminal and cecal contents in Guanling yellow cattle

**DOI:** 10.3389/fmicb.2023.1171563

**Published:** 2023-09-18

**Authors:** Chun Song, Tiantian Zhang, Duhan Xu, Mingming Zhu, Shihui Mei, Bijun Zhou, Kaigong Wang, Chao Chen, Erpeng Zhu, Zhentao Cheng

**Affiliations:** College of Animal Science, Guizhou University, Guiyang, China

**Keywords:** dried distillers’ grains with solubles, Guanling yellow cattle, rumen, cecum, 16S rDNA gene sequencing, metabolomics

## Abstract

Dried distillers’ grains with solubles (DDGS) are rich in nutrients, and partially alternative feeding of DDGS effectively reduces cost of feed and improves animals’ growth. We used 16S rDNA gene sequencing and LC/MS-based metabolomics to explore the effect of feeding cattle with a basal diet (BD) and a Jiang-flavor DDGS diet (replaces 25% concentrate of the diet) on microbiome and metabolome of ruminal and cecal contents in Guanling yellow cattle. The results showed that the ruminal and cecal contents shared the same dominance of *Bacteroidetes*, *Firmicutes* and *Proteobacteria* in two groups. The ruminal dominant genera were *Prevotella_1*, *Rikenellaceae_RC9_gut_group*, and *Ruminococcaceae_UCG-010*; and the cecal dominant genera were *Ruminococcaceae_UCG-005*, *Ruminococcaceae_UCG-010*, and *Rikenellaceae_RC9_gut_group*. Linear discriminant analysis effect size analysis (LDA > 2, *P* < 0.05) revealed the significantly differential bacteria enriched in the DDGS group, including *Ruminococcaceae_UCG_012*, *Prevotellaceae_UCG_004* and *Anaerococcus* in the ruminal contents, which was associated with degradation of plant polysaccharides. Besides, *Anaerosporobacter*, *Anaerovibrio*, and *Caproiciproducens* in the cecal contents were involved in fatty acid metabolism. Compared with the BD group, 20 significantly different metabolites obtained in the ruminal contents of DDGS group were down-regulated (*P* < 0.05), and based on them, 4 significantly different metabolic pathways (*P* < 0.05) were enriched including “Linoleic acid metabolism,” “Biosynthesis of unsaturated fatty acids,” “Taste transduction,” and “Carbohydrate digestion and absorption.” There were 65 significantly different metabolites (47 were upregulated, 18 were downregulated) in the cecal contents of DDGS group when compared with the BD group, and 4 significantly different metabolic pathways (*P* < 0.05) were enriched including “Longevity regulating pathway,” “Bile secretion,” “Choline metabolism in cancer,” and “HIF-1 signaling pathway.” Spearman analysis revealed close negative relationships between the top 20 significantly differential metabolites and *Anaerococcus* in the ruminal contents. Bacteria with high relevance to cecal differential metabolites were *Erysipelotrichaceae_UCG-003*, *Dielma*, and *Solobacterium* that affect specific metabolic pathways in cattle. Collectively, our results suggest that feeding cattle with a DDGS diet improves the microbial structure and the metabolic patterns of lipids and carbohydrates, thus contributing to the utilization efficiency of nutrients and physical health to some extent. Our findings will provide scientific reference for the utilization of DDGS as feed in cattle industry.

## 1. Introduction

Guizhou Moutai liquor is a well-known Jiang-flavor Chinese spirits made from local high-quality glutinous sorghum. The output of Moutai distillers’ grains (DG) exceeds 150,000 tons per year, which contain high water content with perishable properties, easily causing environmental pollution without proper disposal (Li M. Y. et al., 2022). DG is rich in crude protein, crude fat, crude fiber, vitamins, essential amino acids, and other nutrients (Zhang P. G. et al., 2022). Evidences have revealed that partially alternative feeding of DG effectively reduces environmental pollution and feed costs. Fresh DG are dried by the drying process to made into dried distillers’ grains with solubles (DDGS), which effectively improve their nutritional properties and extend their shelf life, and eventually can be added to livestock feed (Wang et al., 2021).

Current studies on the feed utilization of DG are mainly focused on the improvement of animal growth and the promotion of milk production and quality. It has been reported that the substitution of forage and soy protein with high-protein DDG effectively improves feed conversion ratio and milk production in Holstein cows (Hubbard et al., 2009). A recent study reports that feeding cattle with a mixed diet containing yellow wine lees both reduces oxidative stress in heat-stressed cattle and increases the antioxidant capacity of milk (Yao et al., 2021). Another study evaluating the effect of a mixed ration with corn DDGS replacing cornmeal on intake, animal performance, and carcass traits in Nellore cattle demonstrates that DDGS increase average daily gain and carcass gain in cattle (Da et al., 2022). And such an effect was also seen in fattening pigs (Li H. et al., 2022). These studies show that feeding the DDGS diets is beneficial to animals’ growth and improves the quality of animals’ products.

The genome of gastrointestinal microorganisms is rich in genes that regulate the metabolism of carbohydrates, amino acids, vitamins, and short-chain fatty acids. The changes of metabolites can reflect the differential expression of relevant genes, and these changes can be used to assess the physiological or pathological status of the organisms (Gill et al., 2006; Paganelli et al., 2022). With the rapid development of multi-omics in recent years, microbiome and metabolomics have been widely applied in animal-associated studies, such as investigation of the responses to ration-induced milk fat inhibition in dairy cattle and demonstration of the effect of dandelion on the rumen metabolome and microbiota in lactating cows (Zeng et al., 2019; Li Y. et al., 2021). However, there are few reports on the effects of a diet of DDGS on the gastrointestinal microbiome and metabolome in cattle. Guanling yellow cattle is a famous local cattle breed of Guizhou Province, China, and it was approved by the Ministry of Agriculture of China to implement geographical indication registration and protection of agricultural products (No. AGI2016-03-1987) in 2016. In this study, 16S rDNA gene sequencing and LC/MS-based metabolomics technology were adopted to explore the effect of feeding with a basal diet and mixed diet with 25% of concentrate replaced by DDGS on microbiome and metabolome of ruminal and cecal contents in Guanling yellow cattle, which will to some extent provide some theoretical basis for further utilization of Moutai liquor DG as a feed resource in livestock production.

## 2. Materials and methods

### 2.1. Ethics statement

Animal breeding, care, and use, as well as sample collection were performed according to the guidelines of the Experimental Animal Ethics Subcommittee of Guizhou University (No. EAE-GZu-2020-E018). All standard procedures concerning animal care and management were taken, and all efforts were made to minimize their suffering throughout the experiment.

### 2.2. Origin and preparation of DDGS

The DGs used in this study were obtained from the Kweichow Moutai Group in Moutai Town, Renhuai, Guizhou, China. The main ingredients of Moutai DG are distilled sorghum and wheat, that are a byproduct of the brewing processes. A drum dryer was used for the drying treatment before feeding. After centrifugation of fresh Moutai DG, the filter residues and filtrate were separated, and the filtrate was evaporated and concentrated, and then mixed and dried together with the filter residues to obtain DDGS with a moisture content of 10–15%.

### 2.3. Animal experiment

Twelve healthy Guanling yellow cattle (227 ± 18 kg, 18 months old) selected from Guizhou Cattle Industry Group Co., Ltd. were used in this study and were pre-tested without Brucella and foot-and-mouth disease virus-O/-A infection. Two experimental groups (containing 6 cattle each) were randomly assigned to one of the two dietary treatments: a basal diet (BD) and a Jiang-flavor DDGS diet replacing 25% of the concentrate by DDGS. The BD was formulated with reference to the nutritional requirement of 300 kg body weight and 1 kg/d average daily gain according to China’s Beef Feeding Standard (NY/T 815-2004), with a forage: concentrate ratio of 60:40 on a dry matter basis. And *Pennisetum sinese* Roxb was added as a forage in the diet. The composition of experimental concentrate is shown in the Supplementary Table 1, and the nutritional levels of two diets are shown in Supplementary Table 2. The cattle were fed at 9:00 and 16:30 every day for 75 days (15 days for adaptation to the experimental diet and 60 days for formal feeding of the experimental diet). During the entire experiment, the cattle drank water freely, and hygiene and daily management were carried out as a matter of routine. At the end of the experiment, 3 experimental cattle in each group were randomly selected for sacrificed via electrical stunning, and the ruminal contents (BD-R and DDGS-R) and cecal contents (BD-C and DDGS-C) were collected. All samples were collected and frozen in liquid nitrogen for 12 h and then transferred to −80°C for storage until subsequent microbiomic and metabonomic analyses.

### 2.4. 16s rDNA gene amplification, sequencing, and data mining

Total genomic DNA was extracted from ruminal and cecal contents of each cattle using a DNeasy PowerSoil kit (QIAGEN NO12888, New York, USA). We used universal primers to augment the V3 and V4 regions of the 16S rDNA gene, and the primer sequences were 343F (5′-TACGGRAGGCAGCAG-3′) and 798R (5′-AGGGTATCTAATCCT-3′). All the polymerase chain reactions (PCR) were performed using a Tks Gflex DNA Polymerase kit (Takara 580BR10905, Kyoto, Japan). Each run included 2 × Gflex PCR Buffer 15 μL, Forward primer (5 pmol/μL) 1 μL, Reverse primer (5 pmol/μL) 1 μL, Template DNA 1 μL, Tks Gflex DNA Polymerase (1.25 U/μL) 0.6 μL, and ddH_2_O 11.4 μL. Amplification condition was set as follows: the pre-degeneration was performed at 94°C for 5 min, then 26 cycles of denaturation (94°C, 30 s), annealing (56°C, 30 s) and elongation (72°C, 20 s) were performed, followed by extension at 72°C for 5 min.

After PCR amplification, all amplicon libraries were sequenced using an Illumina MiSeq PE 300 platform (Illumina, California, USA). The quality control of each dataset was performed using Trimmomatic v0.35 software to trim the 3′-end of reads and 5′-end of reads, cut low-quality bases (quality scores < 20), and remove short reads (< 50 bp) and “N” records. Paired-end reads were merged into tags using FLASH v1.2.11 with a minimum overlap of a 10-base sequence. UCHIME v2.4.2 was used to remove the chimera sequence in the sequence to obtain final clean reads. Tags were clustered into operational taxonomic units (OTU) at a 97% similarity threshold using Vsearch v1.9.6 software. Species annotation analysis was conducted using RDP classifier v2.2 software (the threshold value was set as 0.7∼1.0). The alpha(α) diversity, including Chao1, Shannon, and Simpson indices was used to determine the richness and diversity of bacterial community. The beta (β)-diversity calculations were performed using QIIME v1.8.0 and displayed with R software v2.15.3. β-diversity was estimated by computing the unweighted UniFrac distance and visualized using principal coordinate analysis (PCoA). Based on Kruskal–Wallis (KW) sum-rank test, the linear discriminant analysis effect size (LEfSe) method was used to identify the most differentially abundant taxonomic features at genus levels. For this analysis, the significance threshold for the Kruskal–Wallis (KW) test was set to 0.05, and the logarithmic linear discriminant analysis (LDA) score cut-off was set to 2.0.

### 2.5. LC/MS-based metabonomic determination and data analysis

The metabonomic analysis was done using an ACQUITY ultra-performance liquid-chromatography (UPLC) I-Class ultra-high performance liquid chromatography tandem VION IMS Q-Tof high-resolution mass spectrometer (MS). 30 mg samples were mixed with L-2-chlorophenylalanine (20 μL, 0.3 mg/mL) and 600 μL cold 20% methanol/water and then vortex-mixed for 2 min. The solution was ultrasonically extracted on ice for 3 min, incubated at −20°C for 30 min and then centrifuged at 13,000×*g* at 4°C for 10 min. A total of 150 μL supernatant was used for LC/MS analysis. Chromatographic separation was carried out with an Acquity uplc BEH C-18 column (100 mm × 2.1 mm, 1.7 μm) with a temperature of 45°C. The UPLC mobile phases were (A) 0.1% formic acid-water and (B) 0.1% formic acid-acetonitrile. The injection volume was 1 μL, and the flow rate was 0.4 mL/min. Mass spectrometry was performed in both positive and negative mode. The ion source temperature was 115°C and the capillary voltage 2.5 kV, the injection voltage was 40 V, the collision voltage was 4 eV, the desolvation temperature was 450 m, the desolvation gas flow was 900 L/h, the mass spectrometry scan range was 50–1,000 amu, scan time was 0.2 s, interscan time 0.02 s. Quality control (QC) sample was prepared by mixing aliquots of each fluid sample. The QC was performed by running four samples as technical replicates in positive and negative mode, respectively, to verify the stability and reproducibility of the UPLC/MS system.

The raw data were used for peak exacting, data baseline filtering and calibration of the baseline, peak alignment, deconvolution analysis, peak identification, and integration of the peak area by the Progenesis QI v2.3. Multidimensional statistical analysis is performed, including the unsupervised Principal Component Analysis (PCA) method and orthogonal partial least squares discriminant analysis (OPLS-DA) with supervised regression modeling to identify the significantly differential metabolites. The differential metabolites were filtered and confirmed by combining the results of the variable importance in the projection (VIP) generated in OPLS-DA, *t*-test (*P* < 0.05) and fold change (FC) of the peak intensities (mean value of peak intensity obtained from DDGS group/mean value of peak intensity obtained from BD group). The screening standard was FC > 1, *P* value < 0.05 and VIP > 1. and KEGG functional annotation and enrichment analysis were performed for differential metabolites. Correlations between differential metabolites (top20) and genus-level microbes were explored with Spearman’s correlation analysis using the OECloud tools in https://cloud.oebiotech.cn. For all analyses, FDR-corrected *P*-values below 0.05 were considered statistically significant.

## 3. Results

### 3.1. 16S rDNA sequencing results and diversity estimates

**FIGURE 1:**
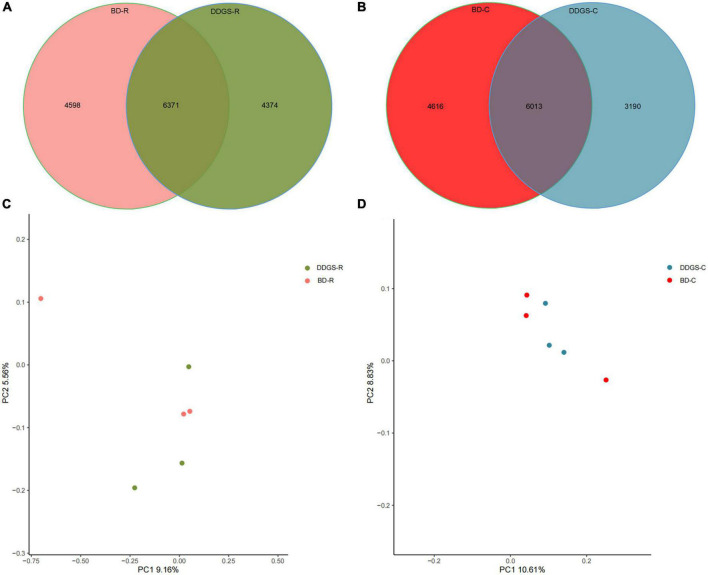
Bacterial diversity analysis. Venn diagram was drawn to illustrate the overlap of microbial OTUs in the ruminal **(A)** and cecal **(B)** contents between the BD and DDGS groups. β-diversity changes in ruminal **(C)** and cecal **(D)** microbiota across BD and DDGS groups were assessed by the principal coordinate analysis (PCoA).

A total of 624,283 clean reads were obtained from 6 rumen fluid samples, and 457,238 clean reads were obtained from 6 cecal samples via Illumina sequencing. Read shear filtration and quality control obtained an average of 66,212 ± 2,976 and 65,266 ± 1,829 in the rumen and cecum, respectively, and the quality control effective rate was over 81%. The Venn diagram showed that the intersection number of OTU was 6,371 in the BD-R and DDGS-R groups (Figure 1A), and the intersection number of OTU was 6,013 in the BD-C and DDGS-C groups (Figure 1B). 4,598 and 4,374 specific OTUs were observed in the BD-R group and DDGS-R group, respectively (Figure 1A). 4,616 and 3,190 specific OTUs were observed in the BD-C group and DDGS-C group, respectively (Figure 1B). As shown in the Supplementary Table 3, no significant differences in Chao1 richness, Shannon diversity index, and Simpson index of rumen and cecum between the BD and DDGS groups was observed. However, the Good’s coverages for all samples exceeded 96%, indicating the accuracy and reproducibility of the sequencing. Based on bacterial species, the PcoA results showed separations between the two treatments in the rumen (Figure 1C) and cecum (Figure 1D), respectively.

### 3.2. Bacterial community composition analyses

**FIGURE 2:**
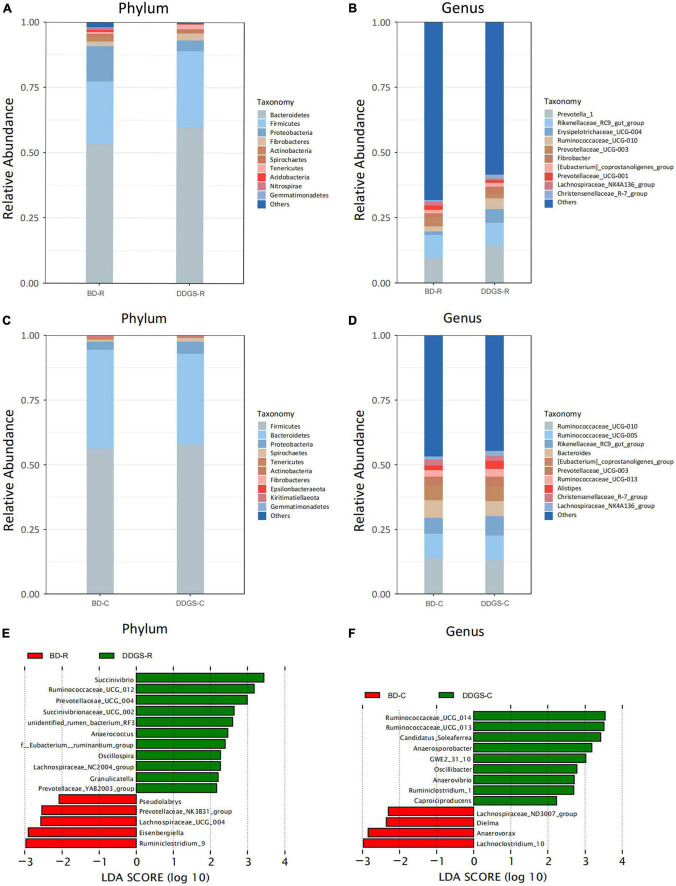
Microbial compositional profiles of the ruminal and cecal contents in the BD and DDGS group. Column chart showing the bacterial composition of the ruminal and cecal contents at the phylum **(A,C)** and genus level **(B,D)**, respectively. Differential microbial genera in the ruminal **(E)** and cecal **(F)** contents of the BD and DDGS groups were tested by linear discriminant analysis effect size (LEfSe) analysis, with linear discriminant analysis (LDA) score of > 2 and *P*-value of < 0.05.

Taxonomic analysis of the reads revealed that *Bacteroidetes, Firmicutes* and *Proteobacteria* were the dominant phyla of the ruminal and cecal contents in two groups (Figures 2A, C). Feeding DDGS increased the abundance of *Bacteroidetes* (*P* = 0.67) and *Firmicutes* (*P* = 0.46) and decreased the abundance of *Proteobacteria* (*P* = 0.35) in the ruminal contents (Figure 2A). However, feeding DDGS decreased the abundance of *Bacteroidetes* (*P* = 0.50) and increased the abundance of *Proteobacteria* (*P* = 0.29) in the cecal contents (Figure 2C). At the genus level, *Prevotella_1* and *Rikenellaceae_RC9_gut_group* were the dominant genera in the ruminal contents (Figure 2B), and *Prevotella_1* (*P* = 0.46), *Erysipelotrichaceae_UCG-004* (*P* = 0.26) and *Ruminococcaceae_UCG-010* (*P* = 0.0.28) were enriched in DDGS-R group. The dominant genera in the cecal contents were *Ruminococcaceae_UCG-010*, *Rikenellaceae_RC9_gut_group*, *Bacteroides* and *Ruminococcaceae_UCG-005*, and *Rikenellaceae_RC9_gut_group* were enriched in DDGS-C group (Figure 2D).

The LEfSe analysis (LDA > 2, *P* < 0.05) performs a non-parametric Kruskal–Wallis (KW) sum-rank test to assess the effect size of each differentially abundant bacterium. At the genus level, the relative abundance of *Succinivibrio*, *Ruminococcaceae_UCG_012*, *Prevotellaceae_UCG_004*, *Anaerococcus*, *Oscillosp-ira*, *Succinivibrionaceae_UCG_002*, *unidentified_rumen_bacterium_RF3*, *f__Eubacterium__ ruminantium_group*, *Lachnospiraceae_NC2004_group*, *Granul-icatella*, and *Prevotellaceae_YAB2003_ group* were increased in the DDGS-C group compared with the BD-C group, while the relative abundance of *Ruminiclostridium_9*, *Eisenbergiella*, *Lachnospiraceae_UCG_004*, *Prevotellaceae_NK3B31_group*, and *Pseudolabrys* were decreased (Figure 2E). The relative abundance of *Ruminococcaceae_ UCG_014*, *Ruminococcaceae_UCG_013*, *Candidatus_Soleaferrea*, *Anaerosporobacter*, *GWE2_31_10*, *Oscillibacter*, *Anaerovibrio*, *Ruminiclostridium_1*, and *Caproiciproducens* were increased in DDGS-C group, however, *Lachnoclostridium_10*, *Anaerovorax*, *Dielma*, and *Lachnospiraceae_ND3007_group* were decreased (Figure 2F).

### 3.3. Metabolomics profiling of ruminal and cecal contents

**FIGURE 3:**
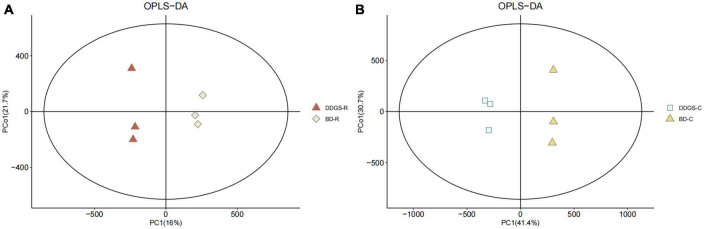
Orthogonal partial least squares discriminant analysis (OPLS-DA) plot of ruminal **(A)** and cecal **(B)** contents metabolites in comparisons between BD and DDGS groups.

In this study, the overlay of the QC samples in the PCA plot indicated that this model was stable, reproducible and consistent for all the samples (Supplementary Figure 1). PCA plots of the ruminal and cecal contents samples provided a less satisfactory separation between the two sets of data (Supplementary Figure 2). Further evaluation of the parameters of the OPLS-DA model presented that the ruminal and cecal fluids of BD and DDGS group had significantly differential metabolite compositions (Figures 3A, B), which indicated that metabolites obtained in the ruminal and cecal fluid samples of two treatments were markedly distinct.

**FIGURE 4:**
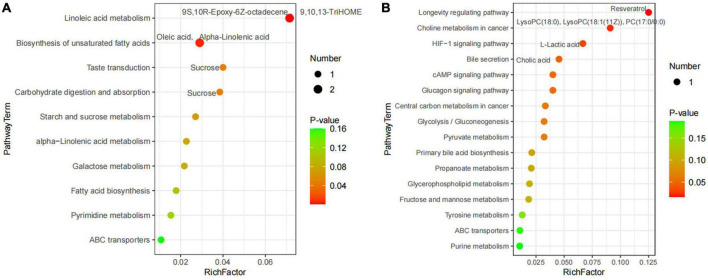
Pathway impact resulting from the differential ruminal **(A)** and cecal **(B)** metabolites in BD and DDGS groups. The X-axis represents pathway impact and the Y-axis represents the pathway enrichment. The larger size of the circle indicates greater pathway enrichment and the darker color indicates higher pathway impact values. The closer the color is to red, the smaller the *P*-value is. Significantly different metabolic pathways are marked around each dot with differential metabolites.

A total of 227 metabolites were identified in the ruminal and cecal metabolome. After *t*-test and VIP filtering for the relative concentrations of metabolites, 20 significantly differential metabolites were downregulated in the DDGS-R group in comparison with the BD-R group (*P* < 0.05, VIP > 1; Table 1). Among them, 11, 2, 2, 2, and 3 metabolites were super-classified into “Lipids and lipid-like molecules,” “Organic oxygen compounds,” “Phenylpropanoids and polyketides,” “Organoheterocyclic compounds,” and “Unclassified,” respectively. These significantly differential metabolites in the rumen were enriched into 10 metabolic pathways, among which “Linoleic acid metabolism,” “Biosynthesis of unsaturated fatty acids,” “Taste transduction,” and “Carbohydrate digestion and absorption” were the significantly differential pathways (*P* < 0.05, Figure 4A).

**TABLE 1:** List of ruminal and cecal fluid metabolites that showed difference between BD and DDGS groups (*P* < 0.05 or *P* < 0.01).

Groups	Metabolites	VIP^1^	*P*-value	FC^2^	Trend
**BD-R vs DDGS-R (*P* < 0.05)**					
	**Lipids and lipid-like molecules**				
	Sophoracoumestan A	1.512	0.010	0.318	Down
	Lonchocarpenin	1.120	0.010	0.244	Down
	9S,10R-Epoxy-6Z-octadecene	2.392	0.011	0.307	Down
	9,10,13-TriHOME	2.100	0.014	0.677	Down
	Didymocalyxin B	1.126	0.020	0.257	Down
	Sideroxylin	1.411	0.029	0.409	Down
	Alpha-Linolenic acid	1.681	0.032	0.378	Down
	9S,10S,11R-trihydroxy-12Z,15Z-octadecadienoic acid	1.703	0.038	0.579	Down
	9,10-Epoxyoctadecenoic acid	1.884	0.038	0.453	Down
	Oleic acid	3.231	0.043	0.337	Down
	Methylliderone	1.853	0.045	0.380	Down
	**Phenylpropanoids and polyketides**				
	7-Hydroxy-2-methylisoflavone	1.096	0.021	0.309	Down
	Cycloartocarpin A	1.914	0.027	0.390	Down
	**Organoheterocyclic compounds**				
	Thymine	2.911	0.015	0.196	Down
	Norfloxacin	1.885	0.026	0.405	Down
	**Organic oxygen compounds**				
	Sucrose	2.946	0.006	0.224	Down
	Erythronic acid	3.069	0.019	0.323	Down
	**Unclassified**				
	MDL 73492 sulfate	4.967	0.024	0.251	Down
	7,2′-Dihydroxy-5,8-dimethyl-4′,5′-methylenedioxyflavan	2.705	0.031	0.451	Down
	2′-Hydroxy-2,4′,6′-trimethoxychalcone	1.668	0.031	0.498	Down
**BD-C vs DDGS-C (*P* < 0.01)**					
	**Lipids and lipid-like molecules**				
	Amorphaquinone	1.592	0.000	2.613	Up
	Pinocembrin 7-O-benzoate	2.602	0.001	13.330	Up
	Naringenin 5,7-dimethyl ether	1.448	0.001	1.669	Up
	8Z-Heptadecen	4.565	0.001	1.503	Up
	Glabratephrinol	2.576	0.002	21.501	Up
	LysoPC [18:1(11Z)]	2.472	0.003	0.145	Down
	Stearoylcarnitine	1.606	0.003	0.122	Down
	10Z-Pentacosene	1.794	0.003	1.421	Up
	1-(O-alpha-D-glucopyranosyl)-27-keto-(1,3R,29R)-triacontanetriol	1.195	0.005	2.173	Up
	**Lipids and lipid-like molecules**				
	21-Methyl-8Z-pentatriacontene	1.489	0.005	1.417	Up
	Euchrenone b3	1.946	0.006	2.217	Up
	1-(O-alpha-D-glucopyranosyl)-3-keto-(1,27R,29R)-triacontanetriol	1.108	0.008	2.035	Up
	Amcinonide	1.184	0.009	0.378	Down
	**Phenylpropanoids and polyketides**				
	Ampelopsin D	8.292	0.002	33.220	Up
	Resveratrol	1.122	0.004	1.797	Up
	**Organic oxygen compounds**				
	8-Nonen-2-one	1.693	0.002	1.610	Up
	2′-Aminoacetophenone	1.402	0.005	1.240	Up
	**Organic acids and derivatives**				
	Serinyl-Methionine	1.229	0.001	4.167	Up
	**Organoheterocyclic compounds**				
	4-Hydroxydebrisoquine	3.977	0.009	1.314	Up
	**Unclassified**				
	5,7,4′-Trimethoxy-4-phenylcoumarin	1.454	0.000	2.750	Up
	Gnetin A	9.321	0.000	15.961	Up
	(2R)-5,4′-Dihydroxy-7-methoxy-6-methylflavanone	1.788	0.001	3.159	Up
	6-Hydroxy-5,7-dimethoxyflavanone	1.076	0.001	2.596	Up
	(25S)-5alpha-cholestan-3beta,4beta,6alpha,8beta,15alpha,16beta,26-heptol	2.184	0.005	1.400	Up
	Canaliculatol	1.364	0.006	2.743	Up
	1,2-Di(2-pyridyl)ethylene	1.353	0.007	6.665	Up
	Fluridone	1.132	0.007	2.806	Up
	7-Hydroxy-3′,4′-dimethoxyflavone	1.651	0.009	3.225	Up

^1^VIP stands for variable importance in projection.

^2^FC (fold change) is calculated as the average level in the DDGS group relative to that in the BD group.

A total of 65 differential metabolites (47 upregulated and 18 downregulated) were obtained in the DDGS-C group compared with the BD-C group (*P* < 0.05, VIP > 1; Supplementary Table 4). Of these, 28 highly significantly differential metabolites were shown in Table 1 (*P* < 0.01 and VIP > 1). Among these 65 differential metabolites, 31, 5, 7, 2, 2, 1, and 17 metabolites were super-classified into “Lipids and lipid-like molecules,” “Phenylpropanoids and polyketides,” “Organoheterocyclic compounds,” “Organic oxygen compounds,” “Organic acids and derivatives,” “Benzenoids,” and “Unclassified,” respectively. These significantly differential metabolites in the cecal contents were enriched into 16 metabolic pathways, among which “Longevity regulating pathway,” “Bile secretion,” “choline metabolism in cancer,” and “HIF-1 signaling pathway” were significantly differential pathways (*P* < 0.05, Figure 4B). These results suggest that the addition of DDGS to the diet of Guanling yellow cattle causes some changes in metabolites and metabolic pathways in the ruminal and cecal contents.

### 3.4. Correlation between the microbiome and metabolome in ruminal and cecal contents

**FIGURE 5:**
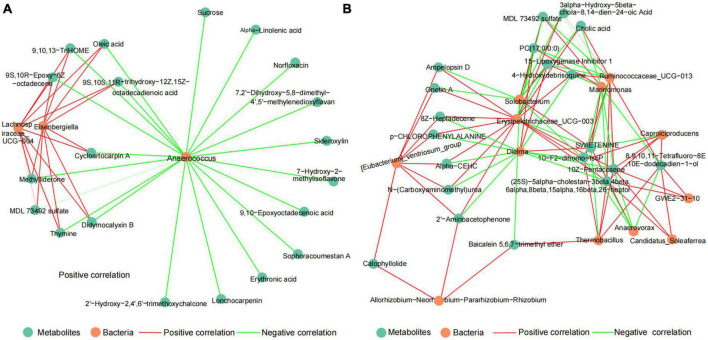
The spearman correlation networks between top20 different metabolites and discrepant bacteria of the ruminal **(A)** and cecal **(B)** contents between BD and DDGS groups. The correlation coefficients with a statistical *P* value < 0.05 and the absolute value > 0.8 were used to build the network graph. Green nodes represent metabolites, orange nodes indicate bacteria. Red lines denote positive correlations, while green lines denote negative correlations.

To explore the potential relationship between microbiota and metabolites of DDGS-induced changes in the rumen and cecum of cattle. The correlation network between discrepant microbiota and top20 differential metabolites in the BD and DDGS groups were assessed based on Spearman’s correlation coefficients (| r| > 0.88, *P* < 0.05). The correlation network of the ruminal consisted of 23 nodes and 38 edges, including 18 positive correlations and 20 negative correlations (Figure 5A). Top 20 differential metabolites, including MDL 73492 sulfate, 9,10,13-TriHOME, Oleic acid, Sucrose, and Erythronic acid were negatively correlated with the relative abundance of *Anaerococcus*, and 9 of them were positively correlated with both *Lachnospiraceae_UCG-004* and *Eisenbergiella*.

Likewise, the correlation network of the cecal contents consisted of 32 nodes and 94 edges, including 58 positive correlations and 36 negative correlations (Figure 5B). It was found that top 20 significantly differential metabolites had highly correlation with the relative abundance of 6 bacteria, including *Erysipelotrichaceae_UCG-003* (13 positive relationships, 4 negative relationships), *Dielma* (3 positive relationships, 9 negative relationships), *Marinomonas* (4 positive relationships, 7 negative relationships), *Ruminococcaceae_UCG-013* (7 positive relationships, 4 negative relationships), Solobacterium (4 positive relationships, 6 negative relationships), and [Eubacterium]_ventriosum_group (8 positive relationships). Furthermore, we identified 12 hub metabolites such as 10-F2-dihomo-IsoP, 10Z-Pentacosene, PC (17:0/0:0), and Cholic acid. These metabolites mainly belong to “Lipids and lipid-like molecules,” and some of these metabolites were “Organic oxygen compounds” and “Unclassified.” Based on correlation network analysis, we found that the abundance of *Anaerococcus* and *Erysipelotrichaceae_UCG-003* may be important for changes in the rumen and cecum metabolites, respectively.

## 4. Discussion

Complex microbiota in gastrointestinal tract of ruminants play critical roles in maintaining homeostasis, regulating energy metabolism, and activating intestinal immunity (Zhang Y. et al., 2021), which can be regulated by changing the diet to improve feed utilization rate, the growth and development of animals (Dill-McFarland et al., 2019; Cui et al., 2022). Our study suggested that DDGS diets have a potent ability to reshape the ruminal and cecal microbial communities. Similar to other studies (Kim et al., 2022), *Bacteroidetes*, *Firmicutes*, and *Proteobacteria* were the dominant phyla in all samples, and the abundance of *Firmicutes* and *Bacteroidetes* were increased in the DDGS-R group. Both *Firmicutes* and *Bacteroidetes* are key participants in fiber fermentation and carbohydrate degradation, producing multiple cellulases to hydrolyze macromolecular compounds such as cellulose and sugars (Chen et al., 2019; Li X. et al., 2021), which facilitates fat deposition, milk fat production and nutrient digestion and absorption (Spence et al., 2006; Brulc et al., 2009; Jami et al., 2014). *Proteobacteria*, whose abundance was increased in DDGS-C group, has been evidenced to be involved in the degradation and fermentation of biopolymers (Zhang Z. et al., 2021). Saccharolytic and anaerobic microbiota (such as *Prevotella_1*, *Ruminococcaceae_UCG-010* and *Fibrobacter*) can especially aid in the degradation of host indigestible carbohydrates (such as cellulose and resistant polysaccharides) into monomeric or dimeric sugars, and subsequently ferment them into short-chain fatty acids (SCFAs) (Tremaroli and Backhed, 2012; Koh et al., 2016). We observed an increase in these bacterial genera in the DDGS-R group. In addition, fiber-degrading bacteria with increased abundance in the DDGS-C group included *Firmicutes*, *Ruminococcaceae_UCG-005*, *Alistipes, Ruminococcaceae_UCG-013, Rikenellaceae_RC9_gut_group*, and *Prevotellaceae_UCG-003*, which exert important fermentative and degradative effects on the indigestible dietary fiber in the foregut (Macfarlane and Macfarlane, 2012; Williams et al., 2016). Similar to other studies (Zou et al., 2020), our study found that the diversity, structure, and composition of the microbial communities were distinct between the rumen and cecum of cattle fed DDGS diet, which also leads to differences in metabolome of rumen and cecum. Similar to ruminal fermentation, in the ruminant’s cecum feed digestion is also performed by a specialized consortia of microorganisms. Fermentable substrates arriving in the cecum are different to those in the rumen, which may result in compositional or structural differences in the microbiota of these two compartments (Popova et al., 2017). These results showed that DDGS diets increased the abundance of bacteria associated with glycolysis and fiber degradation, which is beneficial to the digestion and absorption of polysaccharides in cattle.

Simultaneously, increasing evidence confirms that gut microbes (for instance, *Prevotellaceae_UCG_004*, *Prevotellaceae_YAB2003_group*, *Anaerovibrio*, *Lachnospiraceae_ NC2004_group, Anaerosporobacter* and *Caproiciproducens)* affect lipid deposition and fatty acid content by altering lipid metabolism (Tremlett et al., 2021; Tang et al., 2022; Zhu et al., 2022). LEfSe analysis in our study also showed that these bacteria were enriched in the DDGS group, and we hypothesized that this may affect fat metabolism in the gastrointestinal tract of experimental cattle in the DDGS group and has a positive role in weight gain of cattle. *Lachnospiraceae_NC2004_group* and *Anaerococcus* play critical roles in fermenting and degrading carbohydrates such as cellulose, pectin, and xylan, as well as producing short-chain fatty acids (Murphy and Frick, 2013; Vacca et al., 2020). Both of them were enriched in the DDGS-R group. In addition, another bacterium *Candidatus_Soleaferrea* up-regulated by DDGS diet has been reported to be involved in intestinal immunity (Zou et al., 2022), suggesting that DDGS diet has the potential to promote gut immunity and health. To sum up, DDGS diet changes the gastrointestinal microbiota of experimental Guanling yellow cattle and subsequently enhances the ability of fermenting and degrading macromolecular compounds, including carbohydrates and lipids, altering lipid metabolism, thereby improving the digestion and absorption of nutrients and promoting intestinal health and weight gain in cattle.

Accumulating evidence shows that changes in structure and function of the intestinal microbiota affect host metabolism and immunity (Muller et al., 2021). Similar to the results of previous studies related to dietary modifications (Tang et al., 2021; Zhang X. et al., 2022), our results showed that metabolites affected by DDGS diet were mainly “Lipids and lipid-like molecules,” “Phenylpropanoids and polyketides,” “Organoheterocyclic compounds,” and “Organic oxygen compounds,” which are all important participants in glycolipid metabolisms and redox reactions (Cohain et al., 2021; Hao et al., 2022). It has been reported that *Anaerococcus* participate in glycolysis and produce butyrate (Donohoe et al., 2012; Rivera-Chávez et al., 2016). Interestingly, we found that the *Anaerococcus* was enriched and negatively correlated with 20 downregulated differential metabolites in the DDGS-R group compared with the BD-R group in the spearman analysis. This finding suggests that abundant *Anaerococcus* may improve the metabolism of certain lipids and carbohydrates. Some previous studies reported that *Erysipelotrichaceae* and *[Eubacterium]_ventriosum_group* are the key bacteria for butyrate production to regulate intestinal immunity (Wei et al., 2018; Liu et al., 2019), and *Dielma* and *Solobacterium* may be involved in the synthesis of short-chain fatty acids and biohydrogenation of fatty acids (Salami et al., 2021; Qiu et al., 2022). In the spearman analysis, we found these bacteria had strong correlation with cecal top20 differential metabolites, which mainly belong to “Lipids and lipid-like molecules” and “Organic oxygen compounds.” These findings indicate that DDGS diet changes the gastrointestinal microbiota, metabolism of lipid and organic oxygen compounds in experimental Guanling yellow cattle.

It is well known that sucrose is broken down into glucose in the rumen and absorbed in small intestine. Here we found that sucrose was downregulated in the rumen of the DDGS group and DDGS diet also affected sugar-related metabolic pathways including “Taste transduction” and “Carbohydrate digestion and absorption.” Lactic acid has been evidenced to be the main gluconeogenic precursor and the major fuel for mitochondrial respiration (Brooks, 2020). Interestingly, L-lactic acid is also induced by DDGS to downregulate cecal sugar-related pathways, such as “Glycolysis/Gluconeogenesis,” “Pyruvate metabolism,” and “Central carbon metabolism in cancer.” This suggests that the DDGS diet may promote the process of gastrointestinal glycolysis and glycosylation, thus facilitating the digestion, absorption and utilization of carbohydrates in the experimental cattle. Rumen microorganisms have been reported to promote the hydrogenation of oleic and α-linolenic acid to stearic acid, a saturated fatty acid and one of the main components of solid fat (Vargas et al., 2020; Van Rooijen et al., 2021). In our study, oleic and α-linolenic acids, which were negatively correlated with *Anaerococcus*, were reduced in the rumen of the DDGS group and affected the “Biosynthesis of unsaturated fatty acids” pathway, indicating that *Anaerococcus* affected by DDGS diet promoted the biohydrogenation of oleic and α-linolenic acids, downregulated the synthesis of unsaturated fatty acids, and promoted fat deposition. Cholic acid, a component of digestive juices, is a key signaling molecule that regulates lipid metabolism and immune function in animals (Zhang H. et al., 2022). Our results showed that cholic acid was upregulated by DDGS diet in the cecal “Bile secretion” and “Primary bile acid biosynthesis” pathways, which indicates that DDGS diet promotes the secretion of cholic acid, thereby facilitating the digestion and absorption of lipids.

Linoleic acid, an essential amino acid, has a positive regulatory effect on the body’s growth and development, and can reduce the risk of cardiovascular and cerebrovascular diseases, and it also has beneficial effects on inflammation and degenerative diseases (Marangoni et al., 2020). 9S,10R-Epoxy-6Z-octadecene and 9,10,13-TriHOME are derivatives of linoleic acid (Hamberg, 1991; Fuchs et al., 2018), and they are reduced in the “Linoleic acid metabolism” pathway in the rumen of the DDGS group, indicating that the DDGS diet inhibits the conversion of linoleic acid, which is potentially beneficial to the growth, development, and physical health of the experimental cattle. Evidence has showed that resveratrol plays a beneficial role in preventing chronic diseases associated with inflammation (Malaguarnera, 2019). Our results showed that resveratrol was upregulated by DDGS diet in the “Longevity regulating pathway,” and may have a positive effect on the prevention of intestinal inflammation. Lysophosphatidylcholine (LysoPC) is a pro-inflammatory lipid and is considered as a key marker in pathological conditions (Ren et al., 2022). Another choline metabolite phosphatidylcholine (PC) can mediates proliferative growth and programmed cell death (Saito et al., 2022). Our study showed that LysoPC (18:0), LysoPC [18:1(11Z)], and PC (17:0/0:0) were downregulated in the DDGS group, and they were involved in “Choline metabolism in cancer” and may have a positive effect on preventing the development of cancer and maintaining physical health.

## 5. Conclusion

In summary, our results suggest that diets containing DDGS modify the microbial structure and specific metabolic patterns of the gastrointestinal tract in Guanling yellow cattle, demonstrated by increasing the abundance of bacteria which can promote glycolysis and fibers-degradable, and by modulating the metabolic patterns of rumen and cecum, including alteration of the compositions of “Lipids and lipid-like molecules,” “Phenylpropanoids and polyketides,” “Organoheterocyclic compounds,” and “Organic oxygen compounds,” as well as changes in the metabolic pathways of glucose metabolism, lipid metabolism, and organic oxygen compounds metabolism. These changes may improve digestion and absorption of the nutrients such as carbohydrates and lipids, and facilitate fat deposition and weight gain of beef cattle. In addition, some microorganisms and metabolic pathways affected by DDGS diet are beneficial to intestinal immunity, potentially have a beneficial effect on growth, development, and physical health of cattle. In summary, our findings will provide some theoretical basis for further utilization of Moutai liquor DG as a feed resource in livestock production.

## Data availability statement

All datasets generated for this study are included in the article/Supplementary material.

## Ethics statement

The animal study was approved by the Experimental Animal Ethics Subcommittee of Guizhou University (No. EAE-GZu-2020-E018). The study was conducted in accordance with the local legislation and institutional requirements.

## Author contributions

CS, EZ, and ZC conceived the study. CS, TZ, DX, and MZ performed the experiments. CS, EZ, ZC, and CC analyzed experimental results and data. BZ, KW, CC, and SM assisted with the animal experiments. CS wrote the manuscript. All authors read and approved the final manuscript.
