# Supplementary material for: Impact of feeding dried distillers’ grains with solubles diet on microbiome and metabolome of ruminal and cecal contents in Guanling yellow cattle
Source: Front Microbiol. 2023 Sep 18;14:1171563. doi: 10.3389/fmicb.2023.1171563 (PMC10543695; doi:10.3389/fmicb.2023.1171563)
Supplement: Supplementary file 1 [file Data_Sheet_1.pdf]

Supplementary Material

1 Supplementary Figures and Tables

1.1 Supplementary Figures

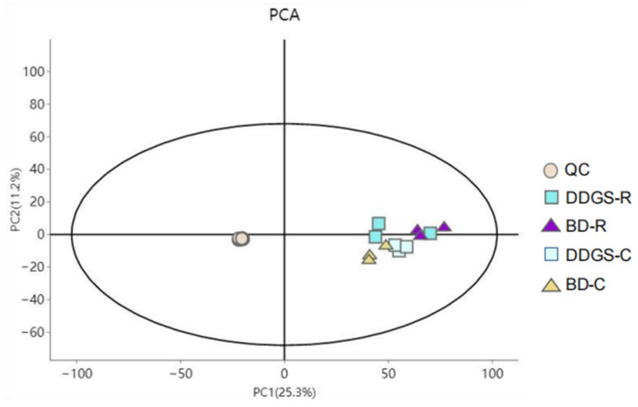

Supplementary Figure 1. Principal component analysis (PCA) of the liquid chromatography/mass spectrometry metabolite profiles of the quality control (QC), ruminal and cecal samples of BD and DDGS group. PC1 = the first principal component; PC2 = the second principal component.

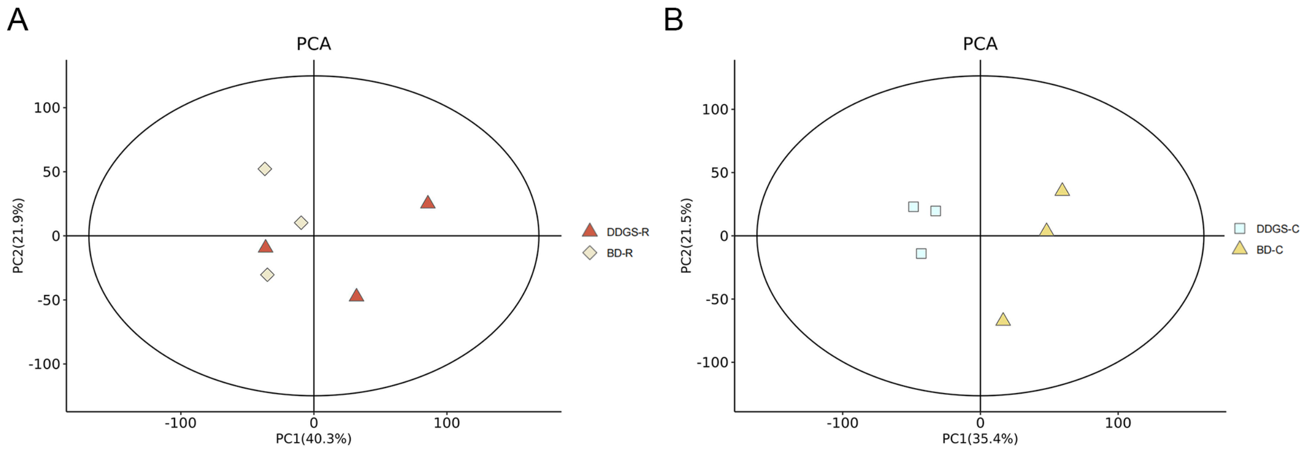

Supplementary Figure 2. Principal component analysis (PCA) of Guanling yellow cattle ruminal (A) and cecal (B) metabolites in comparisons of the BD and DDGS groups, respectively.

1.2 Supplementary Tables

Supplementary Table 1 The composition of concentrates in the diet in the BD group and DDGS group (Dry matter).

.

Item	BD	DDGS
**Ingredient, %**		
Dried distillers' grains with solubles	0	25.0
Ground corn	52.1	42.0
Wheat bran	17.2	10.0
Soybean meal	16.0	10.8
Rapeseed meal	10.8	8.30
Premixes	0.10	0.10
Salt	1.00	1.00
Calcium bisulfate	0.50	0.50
Sodium bicarbonate	1.00	1.00
Rock fine	1.20	1.20
Mold Remover	0.10	0.10
Total	100	100

Supplementary Table 2 The nutritional levels of diets in the BD group and DDGS group (Dry matter).

Item	BD group concentrates	DDGS group concentrates	***Pennisetum Sinese* Roxb**
**Nutritional level,** %			
Dry matter	87.35	87.77	46.95
Gross energy (MJ/kg)	17.23	17.06	16.39
Crude protein	17.65	17.94	4.37
Neutral detergent fiber	28.20	28.58	69.74
Acid detergent fiber	5.47	5.25	45.35
Total P	0.32	0.35	0.78
Total K	0.68	0.67	1.37
Calcium	0.74	0.76	0.59

Supplementary Table 3 The richness and diversity of microorganisms in ruminal and cecal concents from each dietary treatment.

Items	Rumen		SEM	*P*-value	Cecum		SEM[5]	*P*-value
	BD-R[1]	DDGS-R[2]			BD-C[3]	DDGS-C[4]		
**Chao1**	7426.67	8056.67	445.48	0.78	8304.49	7831.35	334.56	0.60
**Shannon**	9.94	9.84	0.07	0.74	9.82	9.69	0.09	0.67
**Simpson**	1	1	0.00	0.23	1	1	0.00	0.78
**Good's coverage（%）**	97	96	0.007	0.74	96	96	0.00	0.67

[1]BD-R, Ruminal contents of test cattle in the BD group.
[2]DDGS-R, Ruminal contents of test cattle in the DDGS group.
[3]BD-C, Cecal contents of test cattle in the BD group.
[4]DDGS-C, Cecal contents of test cattle in the DDGS group.
[5]SEM, standard error of the mean.

Supplementary Table 4 List of ruminal and cecal fluid metabolites that showed significant difference between BD and DDGS groups ($P < 0.05$)

Groups	Metabolites	VIP[1]	*P*-value	FC[2]	Trend
**BD-R vs DDGS-R**					
**Lipids and lipid-like molecules**					
	Sophoracoumestan A	1.512	0.010	0.318	Down
	Lonchocarpenin	1.12	0.010	0.244	Down
	9S,10R-Epoxy-6Z-octadecene	2.392	0.011	0.307	Down
	9,10,13-TriHOME	2.1	0.014	0.677	Down
	Didymocalyxin B	1.126	0.020	0.257	Down
	Sideroxylin	1.411	0.029	0.409	Down
	Alpha-Linolenic acid	1.681	0.032	0.378	Down
	9S,10S,11R-trihydroxy-12Z,15Z-octadecadienoic acid	1.703	0.038	0.579	Down
	9,10-Epoxyoctadecenoic acid	1.884	0.038	0.453	Down
	Oleic acid	3.231	0.043	0.337	Down
	Methylliderone	1.853	0.045	0.38	Down
	**Phenylpropanoids and polyketides**				
	7-Hydroxy-2-methylisoflavone	1.096	0.021	0.309	Down
	Cycloartocarpin A	1.914	0.027	0.39	Down
	**Organoheterocyclic compounds**				
	Thymine	2.911	0.015	0.196	Down
	Norfloxacin	1.885	0.026	0.405	Down
	**Organic oxygen compounds**				
	Sucrose	2.946	0.006	0.224	Down
	Erythronic acid	3.069	0.019	0.323	Down
	**Unclassified**				
	MDL 73492 sulfate	4.967	0.024	0.251	Down
	7,2'-Dihydroxy-5,8-dimethyl-4',5'-methylenedioxyflavan	2.705	0.031	0.451	Down
	2'-Hydroxy-2,4',6'-trimethoxychalcone	1.668	0.031	0.498	Down
**BD-C vs DDGS-C**					
	**Lipids and lipid-like molecules**				
	Amorphaquinone	1.592	0.000	2.613	Up
	Pinocembrin 7-O-benzoate	2.602	0.001	13.330	Up
	Naringenin 5,7-dimethyl ether	1.448	0.001	1.669	Up
	8Z-Heptadecene	4.565	0.001	1.503	Up
	Glabratephrinol	2.576	0.002	21.501	Up
	10Z-Pentacosene	1.794	0.003	1.421	Up
	1-(O-alpha-D-glucopyranosyl)-27-keto-(1,3R,29R)-triacontanetriol	1.195	0.005	2.173	Up
	(21-Methyl-8Z-pentatriacontene	1.489	0.005	1.417	Up
	Euchrenone b3	1.946	0.006	2.217	Up
	1-(O-alpha-D-glucopyranosyl)-3-keto-(1,27R,29R)-triacontanetriol	1.108	0.008	2.035	Up
	Sideroxylin	1.144	0.011	1.903	Up
	Calophyllolide	2.860	0.014	2.032	Up
	Eicosanoyl-EA	1.724	0.015	1.853	Up

Supplementary Table 4 List of ruminal and cecal fluid metabolites that showed significant difference between BD and DDGS groups (P < 0.05) (continued).

Groups	Metabolites	VIP[1]	*P*-value	FC[2]	Trend
**BD-C vs DDGS-C**					
	**Lipids and lipid-like molecules**				
	1b,3a,12a-Trihydroxy-5b-cholanoic acid	1.262	0.020	2.658	Up
	14S-Methyl-1-octadecene	1.561	0.023	1.562	Up
	Glyceryl 5-hydroxydecanoate	1.653	0.024	1.622	Up
	Scandinone	1.772	0.029	1.893	Up
	10-F2-dihomo-IsoP	3.902	0.032	1.524	Up
	Tricetin 3'-methyl ether 7,5'-diglucuronide	1.287	0.038	1.336	Up
	Cholic acid	7.684	0.038	2.305	Up
	Methylliderone	1.140	0.041	1.508	Up
	N-(6-aminohexanoyl)-6-aminohexanoic acid	1.329	0.042	2.695	Up
	8,9,10,11-Tetrafluoro-8E,10E-dodecadien-1-ol	2.314	0.043	1.473	Up
	Stearoylcarnitine	1.606	0.003	0.122	Down
	LysoPC(18:1(11Z))	2.472	0.003	0.145	Down
	amcinonide	1.184	0.009	0.378	Down
	2-O-(beta-D-galactopyranosyl-(1->6)-beta-D-galactopyranosyl) 2S-hydroxyundecanoic acid	1.860	0.015	0.248	Down
	Didymocalyxin B	1.336	0.037	0.426	Down
	PC(17:0/0:0)	6.425	0.047	0.462	Down
	**Phenylpropanoids and polyketides**				
	Ampelopsin D	8.292	0.002	33.220	Up
	Resveratrol	1.122	0.004	1.797	Up
	p-CHLOROPHENYLALANINE	6.166	0.016	1.540	Up
	Rosmarinic acid	1.290	0.019	1.410	Up
	Baicalein 5,6,7-trimethyl ether	2.703	0.031	2.715	Up
	Organic oxygen compounds				
	8-Nonen-2-one	1.693	0.002	1.610	Up
	2'-Aminoacetophenone	1.402	0.005	1.240	Up
	**Organic acids and derivatives**				
	Serinyl-Methionine	1.229	0.001	4.167	Up
	L-Lactic acid	1.053	0.043	0.364	Down
	**Organoheterocyclic compounds**				
	4-Hydroxydebrisoquine	3.977	0.009	1.314	Up
	Alpha-CEHC	2.716	0.017	1.619	Up
	Oxindole	1.845	0.035	5.431	Up
	Capsicum annuum Fluorescent chlorophyll catabolite	1.486	0.019	0.242	Down
	Alosetron	1.219	0.036	0.574	Down
	Uric acid	2.103	0.044	0.147	Down
	Hematoporphyrin	1.264	0.046	0.528	Down

Supplementary Table 4 List of ruminal and cecal fluid metabolites that showed significant difference between BD and DDGS groups ($P < 0.05$) (continued).

Groups	Metabolites	VIP[1]	*P*-value	FC[2]	Trend
**BD-C vs DDGS-C**					
	**Benzenoids**				
	Phthalic acid	1.153	0.014	1.977	Up
	**Unclassified**				
	5,7,4'-Trimethoxy-4-phenylcoumarin	1.454	0.000	2.750	Up
	Gnetin A	9.321	0.000	15.961	Up
	(2R)-5,4'-Dihydroxy-7-methoxy-6-methylflavanone	1.788	0.001	3.159	Up
	6-Hydroxy-5,7-dimethoxyflavanone	1.076	0.001	2.596	Up
	(25S)-5alpha-cholestan-3beta,4beta,6alpha,8beta,15alpha,16beta,26-heptol	2.184	0.005	1.400	Up
	Canaliculatol	1.364	0.006	2.743	Up
	1,2-Di(2-pyridyl)ethylene	1.353	0.007	6.665	Up
	Fluridone	1.132	0.007	2.806	Up
	7-Hydroxy-3',4'-dimethoxyflavone	1.651	0.009	3.225	Up
	Copalliferol B	1.639	0.016	2.150	Up
	3alpha-Hydroxy-5beta-chola-8,14-dien-24-oic Acid	10.561	0.026	2.410	Up
	N-(Carboxyaminomethyl)urea	4.269	0.028	1.525	Up
	1-(3,4-Dihydroxyphenyl)-1-decene-3,5-dione	1.897	0.011	0.304	Down
	15-Lipoxygenase Inhibitor 1	6.878	0.011	0.473	Down
	Dihydroisolysergic acid II	1.787	0.013	0.394	Down
	MDL 73492 sulfate	5.818	0.035	0.347	Down
	SWIETENINE	7.291	0.038	0.293	Down

[1]VIP stands for variable importance in projection.
[2]FC (fold change) is calculated as the average level in the DDGS group relative to that in the BD group.
